# Distinguishing physical mechanisms using GISAXS experiments and linear theory: the importance of high wavenumbers

**DOI:** 10.1038/s41598-017-01059-x

**Published:** 2017-05-17

**Authors:** Scott A. Norris, Joy C. Perkinson, Mahsa Mokhtarzadeh, Eitan Anzenberg, Michael J. Aziz, Karl F. Ludwig

**Affiliations:** 1 Department of Mathematics, Southern Methodist University, Dallas Texas, 75275 USA; 2 Harvard School of Engineering and Applied Sciences, Cambridge Massachusetts, 02138 USA; 3 Department of Physics, Boston University, Boston Massachusetts, 02215 USA; 4 Division of Materials Science and Engineering, Boston University, Boston Massachusetts, 02215 USA

## Abstract

In this work we analyze GISAXS measurements of the structure factor of Si surfaces evolving during 1 keV Ar+ ion bombardment. Using newly-developed methods sensitive to the full range of experimentally-available wavenumbers *q*, we extract the linear amplification rate *R*(*q*) governing surface stability over a range of wavenumbers 4–5 times larger than has previously been obtained. Comparing with theoretical models also retaining full wavenumber-dependence, we find an excellent fit of the experimental data over the full range of irradiation angles and wavenumbers. Moreover, the fitted parameter values represent experimental evaluation of the magnitudes of most physical mechanisms currently believed to be important to the pattern-formation process. In all cases, the extracted values agree well with direct observations or atomistic simulations of the same quantities, suggesting that GISAXS analysis may allow more powerful comparison between experiment and theory than had previously been thought.

## Introduction

Energetic ions and atoms play an important role in a wide variety of surface and thin film processes including plasma etching, sputter deposition, and ion beam assisted deposition. At low energy irradiation, where the effect of the ions is confined primarily to the surface, a variety of surface modification phenomena are observed, including ultra-smoothening on the one hand^1^, or alternatively, the spontaneous formation of arrays of nanostructures, with lengths as small as 10 nm^2^. Because ion bombardment is already ubiquitous in industrial settings, and is relatively inexpensive compared to other surface processing techniques, self-organized patterning by ion bombardment could enable a simple, economical means of inducing well-defined nanoscale structures in a variety of settings. Understanding the fundamental behavior of surfaces during ion bombardment is therefore a vital goal; however, despite many years of research on the topic, a predictive understanding of such structure formation has proved elusive.

The goal of most theoretical studies into ion-induced pattern formation is to understand the evolution of the surface height $h({\bf{r}},t)=h(x,y,t)$ – where *x* corresponds to the in-plane projection of the ion direction, *y* is the in-plane direction perpendicular to *x*. A conceptually powerful approach is to focus on very early times during pattern development, when the amplitude of undulations in the surface height are very small, and the evolution is well-approximated by a linear first-order differential equation of the form:

1 $$\frac{\partial \tilde{h}({\bf{q}},t)}{\partial t}=R({\bf{q}})\tilde{h}({\bf{q}},t)+\beta ({\bf{q}},t)$$

where $\tilde{h}({\bf{q}},t)$ is the Fourier transform of $h({\bf{r}},t)$, the linear coefficient *R*(**q**) is called the *amplification factor* or *dispersion relation*, and the constant *β*(**q**, *t*) is the Fourier transform of a stochastic noise. A positive *R*(**q**) at a given bombardment angle drives exponential amplification of modes of wavevector **q** leading to topographic instability. Conversely a negative *R*(**q**) dampens fluctuations and stabilizes modes of wavevector **q**, leading to smoothing^3^.

The linear framework represented by Eq. (1) becomes especially powerful when coupled to GISAXS measurements of surface scattering, which correlate to the surface structure factor *S*(**q**, *t*) when the surface structure amplitudes are small. As described in prior work^4, 5^, the early-time assumption that surface dynamics are governed by Eq. (1) leads to a solution governing the evolution of the structure factor of the form

2 $$\langle S({\bf{q}},t)\rangle =[S(q,0)+\frac{\alpha }{2R({\bf{q}})}]\,\exp [2R({\bf{q}})t]-\frac{\alpha }{2R({\bf{q}})}$$

where *α* is the amplitude of the noise. By fitting the experimentally-measured timeseries *S*(**q**, *t*) for each wavenumber *q* to Eq. (2), the dispersion relation *R*(*q*) can be reconstructed, and then compared directly to theoretical models of early surface morphology dynamics, in principle providing a direct connection between theory and experiment. This approach was used previously to compare the relative contributions of sputtered vs redistributed atoms during ion bombardment^4, 5^.

However, despite their contributions, these initial explorations were constrained by a focus on small wavenumbers |**q**|. Experimentally, ion-induced nanostructures often have characteristic wavelengths *λ* larger than the thickness *h*_0_ of a thin amorphous film that develops atop an irradiated crystalline semiconductor. This naturally suggests a characteristic dimensionless number *Q* = *h*_0_ *q* that is limited in its magnitude. In GISAXS experiments, the limited range of *Q* means that the structure factor *S*(**q**, *t*) has the greatest intensity and variation at small wavenumbers, and refs 4, 5 focused on a range of $q\in [0.1,0.4]\,{{\rm{nm}}}^{-1}$ considerably smaller than the full range $q\in [0.1,1.5]\,{{\rm{nm}}}^{-1}$ recorded by the detector. In theoretical models, the limited range of *Q* leads to a common simplification called the *longwave approximation*, in which the amplification factor *R*(**q**) is expanded as a Taylor series about *q* = 0. For pure materials this leads to approximations of the form

3 $$R({\bf{q}})\approx -\,{S}_{x}(\theta ){q}_{x}^{2}-{S}_{y}(\theta ){q}_{y}^{2}-B(\theta ){|{\bf{q}}|}^{4},$$

where *S*_*x*,*y*_(*θ*) are irradiation angle-dependent coefficients for the curvature-dependent surface response in the two directions, and *B*(*θ*) is the coefficient of an unconditionally-stabilizing surface energy relaxation mechanism (usually presumed to be isotropic), that is required to regularize the system in the case that ${S}_{X,Y}(\theta ) < 0$.

Although useful in the development of physical intuition, the longwave approximation suffers from two very important drawbacks in the context of experimental hypothesis testing with GISAXS. First, the approximation (3) *is quantitatively poor* as *Q* increases. For example, in a typical experiment from recent studies on Si with 1 keV Ar^+^ bombardment, we observed at *θ* = 65° the formation of ripples with a wavenumber of about $q\approx 0.28\,{{\rm{nm}}}^{-1}$ ^4^, on a thin film with a thickness of approximately 6 nm^6^. The resulting value of $Q\approx 1.7$ is greater than unity, and therefore calls into question the validity of the truncated expansion (3). Second, and more perniciously, a focus on small wavenumbers *hinders the distinguishing of different physical effects*. Many physical processes occur simultaneously during ion bombardment, including (a) the sputtering of some surface atoms^7–9^, (b) the relocation of many others^1, 10, 11^, (c) surface diffusion^9, 12^, (d) the creation of stress within the film^13–17^, and (e) the relaxation of strain and surface energies via viscous flow^18–20^. Critically, all of these processes lead to longwave linear growth rates with the same form exhibited in Eq. (3), making an experimental estimation of the relative magnitudes of different effects very difficult. Only at higher values of *Q* will different physical mechanisms be readily distinguished.

We here present a study dedicated to the comprehensive consideration of the high-*Q* regime, asking (a) how best to obtain and analyze the full range of experimentally-available data to evaluate *R*(*Q*) at high values of *Q*, (b) whether models retaining full *Q*-dependence will provide a significantly better fit to the resulting values, and (c) whether these new fits enable us to distinguish different contributions to the fundamental physics of ion-induced pattern formation. Our principal findings are fourfold:

1. With careful attention to noise reduction in both the experimental and analytical stages, it is possible to extract meaningful measurements of the dispersion relation that extend to high values of *Q*.
2. It is only possible to fit the resulting amplification rates using theoretical models retaining full *Q*-dependence; therefore such models should be preferred for all tasks involving quantitative comparison with experiment.
3. Fits of better models to broader data allows evaluation of more model parameters than has previously been possible; indeed, we obtain reasonable values of the magnitudes of *most mechanisms of interest*.
4. There is significant evidence that ion-induced stress may play an equal or greater role in ion-induced pattern formation than competing mechanisms such as erosion and impact-induced redistribution.

These findings, which represent the first direct experimental comparison between the relative magnitude of various physical mechanisms operating during ion-bombardment of pure materials, suggest that GISAXS analysis may represent a considerably more powerful tool for the comparison of theory and experiment than had previously been thought. We therefore include as many details of our approach as possible to facilitate future efforts in this direction. Moreover, we release the software we developed to perform our analyses as an open-source library.

## Results

Using the methods described below in the Methods section, we obtained our primary results in three stages. First, we performed GISAXS measurements of the structure factor *S*(**q**, *t*) associated with early times during the irradiation process. Second, using a newly-developed statistical approach, we fit the parameters in Eq. (2) to the resulting data to extract values of *R*(*q*_*x*_, 0) and *R*(0, *q*_*y*_) for a variety of angles of incidence, over a much wider range of *q* than has previously been obtained. Third, we used the extracted *R*(*q*) to fit the parameters in a composite model containing the three most widely-studied physical mechanisms known to operate during ion-induced pattern formation on amorphous, monotomic targets:

4 $$\begin{array}{ccc}R(q) & = & -\frac{\gamma {\eta }^{-1}(\theta )}{2{h}_{0}}(\frac{Q[\sinh (2Q)-2Q]}{1+2{Q}^{2}+\cosh (2Q)})\\  &  & -6fA{C}_{X,Y}^{{\rm{s}}{\rm{t}}{\rm{r}}{\rm{e}}{\rm{s}}{\rm{s}}}(\theta )\frac{{Q}^{2}}{[1+2{Q}^{2}+\cosh (2Q)]}\\  &  & -{C}_{X,Y}^{{\rm{d}}{\rm{i}}{\rm{s}}{\rm{p}}{\rm{l}}.}(\theta )(1-\exp (\,-\,\frac{1}{2}{(Dq)}^{2})).\end{array}$$

In this composite model, the first term on the right-hand side is Orchard’s result for viscous flow due to surface energy relaxation^18^, containing the surface energy *γ*, the fluidity ${\eta }^{-1}$, and amorphous film thickness *h*_0_. The second term is a result describing viscous flow due to an ion-induced stress^21^, containing the flux *f*, a measure *A* of the stress generated per ion impact, and a shape function ${C}_{X,Y}^{{\rm{stress}}}(\theta )$ that can differ in the *x*- and *y*-directions. The third term is a simplification of Bradley’s result for the effect of ion-induced sputtering^22^, containing another shape function ${C}_{X,Y}^{{\rm{displ}}{\rm{.}}}(\theta )$ and a characteristic lengthscale *D*. This composite model omits surface diffusion (discussed below), but is otherwise an essentially complete aggregate of known mechanisms.

**Figure 1:**
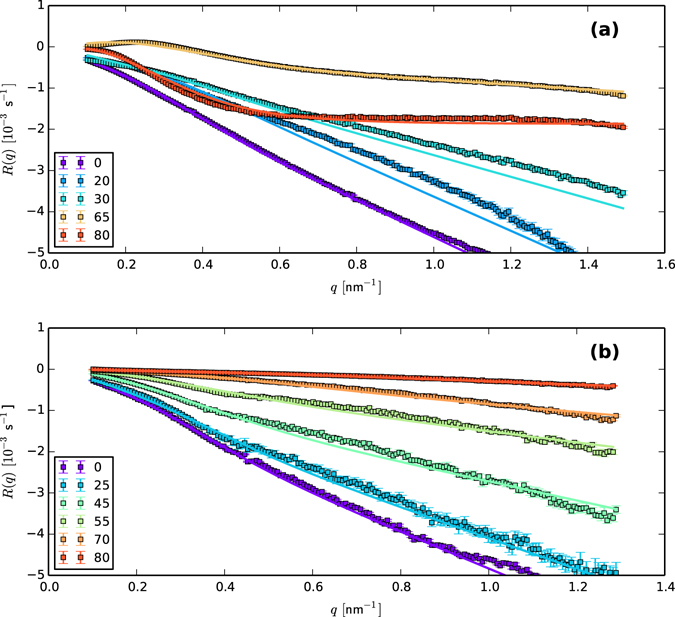
Results of analyzing GISAXS data over an extended range of *q* (squares), together with fits of that data to models retaining full wavenumber dependence (curves). Shown are (**a**) evaluations of *R*(*q*_*x*_, 0) for irradiation angles $\theta \in [0,20,35,45,65,80]$ over the range $q\in [-1.5,1.5]\,{{\rm{nm}}}^{-1}$, and (**b**) evaluations of *R*(0, *q*_*y*_) for irradiation angles of $\theta \in [0,25,45,55,70,80,85]$ over the range $q\in [-1.25,1.25]\,{{\rm{nm}}}^{-1}$.

The extracted *R*(*q*), and the fitted composite models, are shown in Fig. (1), where good agreement between experiment and model is observed. Of course, the total number of parameters in the model means that the significance of the fit quality depends on whether or not the fitted values of the parameters match up with external estimates where such are available, or at least with general expectations where external estimates are unavailable. Therefore, we now turn to a more detailed consideration of the angle-dependence of the fitted parameter values. For consistency with previously published results studying long-wavelength approximations, and comparisons between mechanisms with the same long-wavelength form and units, we will discuss, in addition to the physical parameters in the problem, the coefficients of the long-wave approximations of each of the three mechanisms just described, defined via

5 $${B}_{{\rm{O}}{\rm{r}}{\rm{c}}{\rm{h}}.}(\theta )=\mathop{{\rm{l}}{\rm{i}}{\rm{m}}}\limits_{q\to 0}\frac{1}{{q}^{4}}\cdot \frac{\gamma }{2\eta {h}_{0}}\frac{Q[\sinh (2Q)-2Q]}{[1+2{Q}^{2}+\cosh (2Q)]}=\frac{\gamma {h}_{0}^{3}(\theta )}{3\eta (\theta )}$$

6 $${S}_{X,Y}^{{\rm{s}}{\rm{t}}{\rm{r}}{\rm{e}}{\rm{s}}{\rm{s}}}(\theta )=\mathop{{\rm{l}}{\rm{i}}{\rm{m}}}\limits_{q\to 0}\frac{1}{{q}^{2}}\cdot \frac{6fA{C}_{X,Y}^{{\rm{s}}{\rm{t}}{\rm{r}}{\rm{e}}{\rm{s}}{\rm{s}}}(\theta ){Q}^{2}}{[1+2{Q}^{2}+\cosh (2Q)]}=3fA{h}_{0}^{2}(\theta ){C}_{X,Y}^{{\rm{s}}{\rm{t}}{\rm{r}}{\rm{e}}{\rm{s}}{\rm{s}}}(\theta )$$

7 $${S}_{X,Y}^{{\rm{displ}}{\rm{.}}}(\theta )=\mathop{\mathrm{lim}}\limits_{q\to 0}\frac{1}{{q}^{2}}\cdot [{C}_{X,Y}^{{\rm{displ}}{\rm{.}}}(\theta )(1-\exp (-\frac{1}{2}{(Dq)}^{2}))]=\frac{1}{2}{D}^{2}{C}_{X,Y}^{{\rm{displ}}{\rm{.}}}(\theta )\mathrm{.}$$

### Film thickness

**Figure 2:**
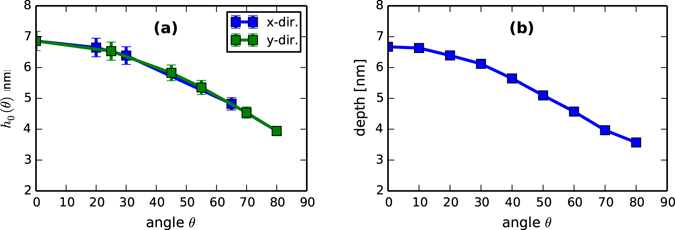
Comparison of (**a**) fitted values of the film depth, to (**b**) values obtained from simulation using TRI3DST. Essentially exact agreement is observed across all angles.

In the ion-enhanced viscous flow conceptualization proposed by Umbach^19^ and adopted by more recent models of stress^21, 23^, viscous flow is confined to a thin surface layer that is provided an enhanced fluidity due to the energy deposited by ions in collision cascades. Sigmund’s prolate Gaussian ellipsoid approximation of energy deposition suggests that the thickness would be a decreasing function of angle^24^, which suggests the form for *h*_0_(*θ*) appearing in Eq. (15). In Fig. 2a, we plot the fitted values of *h*_0_(*θ*), which do indeed exhibit a decreasing trend, from around 6.5 nm to around 3 nm as *θ* increases.

Although these values seem reasonable, they can readily be compared to simulations of ion impact using atomistic simulations. Using TRI3DST^25^ via the PyCraters library^26^, we performed 1000 simulations of 1000 eV Ar^+^ → Si at each of 9 angles. As a measure of the film depth, we take the mean plus two standard deviations of the ion implantation distribution (see e.g., ref. 24; this approach produces very good agreement with limited experimental data available in ref. 27). The results are plotted in Fig. 2b. Comparing to final results for the fitted film depth in Fig. 2a, we find very good agreement between fit and simulation.

### Fluidity

**Figure 3:**
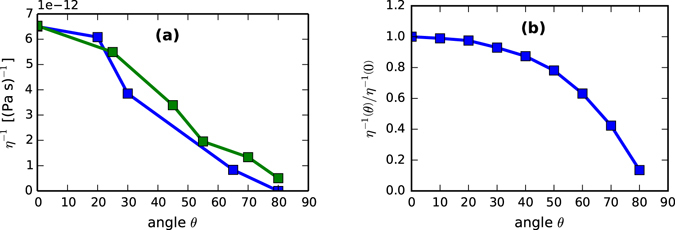
(**a**) Fitted values of the fluidity ${\eta }^{-1}(\theta )$. We note the decrease toward zero as *θ* → 90° (as expected of a flux-driven mechanism), and the reasonable agreement between coefficient values in the *x* and *y* directions (as expected of an isotropic mechanism). (**b**) Comparison to simulated values of the model in Eq. (8) (scaled to show shape rather than absolute magnitude).

We now turn to the effective fluidity parameter ${\eta }^{-1}$. Within the ion-enhanced viscous flow conceptualization, this fluidity is assumed to be proportional to the deposited power through the ion flux. Because the flux through a plane normal to the surface decreases like cos (*θ*), we might expect the fitted value of ${\eta }^{-1}$ to decrease with increasing *θ*. Moreover, because the viscous flow mechanism is isotropic, we should expect the fitted value to be the same regardless of the direction from which it is measured. Fitted values of ${\eta }^{-1}$ are shown in Fig. 3a, where both expectations are confirmed. (We note that both properties can also be observed directly from the *R*(*q*) curves in Fig. 1, whose slopes in the limit $q\to \infty$ of Eq. (4) are seen to be proportional to $\gamma /\eta$). These findings are intriguing, because although viscous flow is increasingly assumed to be the dominant relaxation mechanism^19^, few estimates of this parameter exist in the literature.

The *magnitude* of our fitted values, represented by ${\eta }^{-1}(0)$, can be compared to the only known estimate of fluidity, found in the supplemental material of ref. 4. There, molecular dynamics simulations in ref. 20 are extrapolated to the present irradiation environment, obtaining an estimate of $\eta \sim 2.5\times {10}^{12}{\rm{P}}{\rm{a}}{\rm{s}}$. Here, because the constants *B*_Orch._ and *h*_0_ are independent fit parameters, they can be used directly to evaluate the viscosity in the thin film:

$$\eta (0)=\frac{\gamma {h}_{0}^{3}(0)}{3{B}_{{\rm{Orch}}{\rm{.}}}(0)}=1.5\times {10}^{11}\,{\rm{Pa}}\,\sec $$

where we have used a surface energy of γ = 1.36 J/m^2^ ^20, 28^. This is within about an order of magnitude of the scaled simulation result obtained in ref. 4; because the latter is, itself, an estimate with some uncertainty, we consider this a reasonable level of agreement.

The *angle*-*dependence* of the fluidity, represented by ${\eta }^{-1}(\theta )/{\eta }^{-1}(0)$, has not received much attention in the literature. We therefore briefly propose a potential model that could be informed by BCA simulations. If one assumes that the viscous relaxation occurs continuously and globally at all points in the film, then the power could simply be divided by the film thickness to obtain the average power density:

8 $${\eta }^{-1}(\theta )\propto \frac{\cos (\theta )E(\theta )}{{h}_{0}(\theta )},$$

where *E*(*θ*) is the deposited energy per impact. Like *h*_0_ above, *E*(*θ*) can be estimated from TRI3DST, by subtracting the energies of sputtered atoms and reflected ions from the incoming ion energy. Simulated values of this function are shown in Fig. 3b, showing reasonable agreement with the fitted values in Fig. 3a. This suggests interesting questions for future efforts to understand the atomistic mechanisms of ion-enhanced fluidity.

### Stress Coefficient

**Figure 4:**
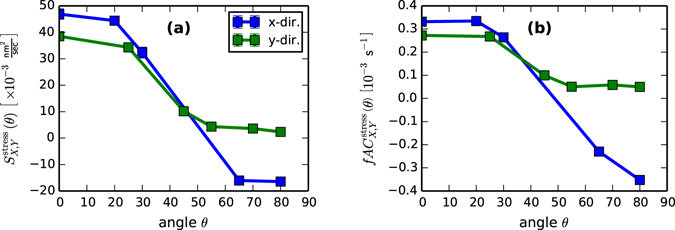
Fitted values of (**a**) the longwave stress coefficient ${S}_{X,Y}^{{\rm{stress}}}(\theta )=3fA{h}_{0}^{2}{C}_{X,Y}^{{\rm{stress}}}$, and (**b**) the rate coefficient $fA{C}_{X,Y}^{{\rm{stress}}}(\theta )$. We note the excellent agreement of the latter with theoretically-predicted forms $\{\cos (2\theta ),{\cos }^{2}(\theta )\}$.

We next turn to the angle-dependent coefficient of stress. For comparison with prior studies using the long-wave approximation, we first report the value of ${S}_{X,Y}^{{\rm{stress}}}(\theta )$ in Fig. 4a. Then, we present the same data in a different form, plotting $fA{C}_{X,Y}^{{\rm{stress}}}(\theta )$ in Fig. 4b. The resulting curves are very consistent with the functional forms predicted in ref. 21: ${C}_{X,Y}^{{\rm{stress}}}(\theta )=\{\cos (2\theta ),{\cos }^{2}(\theta )\}$. Therefore, we may use them to make a *direct experimental evaluation* of the parameter group $fA\approx 3\times {10}^{-4}\,{{\rm{s}}}^{-1}$. We note that the agreement with theory is *not* apparent in the longwave limit – Because the longwave coefficient ${S}_{X,Y}^{{\rm{stress}}}$ contains *h*_0_, which itself depends on θ, it has a different angular dependence than the parameter group $fA{C}_{X,Y}^{{\rm{stress}}}(\theta )$.

As an external test of these fitted values, we may use them to predict the stress in the amorphous film, which can be compared to a direct experimental observation using wafer curvature measurements. On the one hand, ref. 21 gives the steady thin film stress in terms of *fA* and $\eta$, which appear in the forms for ${C}_{{\rm{stress}}}^{LW}$ and ${C}_{{\rm{Orchard}}}^{LW}$. This allows a translation from fitted coefficients to implied steady stress:

9 $${\parallel {{\bf{T}}}_{0}\parallel }_{{\rm{t}}{\rm{h}}{\rm{e}}{\rm{o}}{\rm{r}}{\rm{y}}}=6fA\eta =\frac{{S}_{X,Y}^{stress}(0)}{{B}_{Orch.}(0)}\cdot \frac{2\gamma {h}_{0}}{3}\approx 0.3{\rm{G}}{\rm{P}}{\rm{a}}.$$

On the other hand, the stress in the film can be inferred directly from wafer curvature measurements. Under normal incidence irradiation at 1 keV, using the same ion source and flux that was used to obtain the amplification factors in Section 6, we have measured the development of a steady-state compressive stress of approximately 0.2–0.3 GPa, in very good agreement with the values inferred from the fits. We note that this value is slightly above the 0.2 GPa attributed in ref. 29 to the amorphization of the thin film.

### Prompt Effects

**Figure 5:**
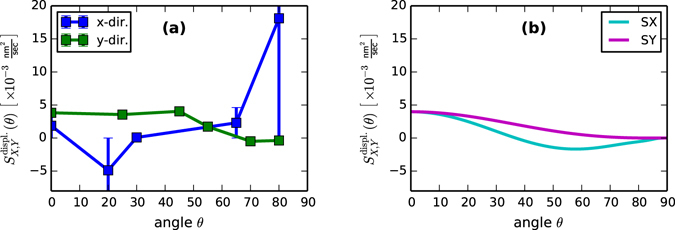
Comparison of coefficients of displacement effects. (**a**) As fitted to Eq. (14) from the values of *R*(*q*). (**b**) As computed from crater function simulations at 18 irradiation angles using PyCraters^26^, using an atomic volume for Silicon of ${\rm{\Omega }}\approx 0.02\frac{{{\rm{nm}}}^{3}}{{\rm{atom}}}$, and a flux of $f\approx 2\times {10}^{12}\frac{{\rm{ions}}}{{{\rm{cm}}}^{2}\,\sec }$ as used at BNL. (﻿Note that the uncertainty intervals in the *x*-direction are quite large, but symmetric. Therefore, to facilitate visual comparison, parts of some error bars are cut off).

We conclude by examining the fitted values of the displacement term, ${S}_{X,Y}^{{\rm{displ}}{\rm{.}}}(\theta )$. These are displayed in Fig. 5a. As an external test of these values, we turn to the “Crater Function Framework,” developed to predict the angle-dependent form of these coefficients directly from the results of atomistic simulation of individual ion impacts (see general results in ref. 30, and subsequent particular results in refs 31 and 32). Using the “PyCraters” Python library^26^, we obtained values of ${S}_{X,Y}^{{\rm{displ}}{\rm{.}}}(\theta )$ for the experimental conditions described above. These are plotted in Fig. 5b.

Comparing these panels, we see that the fitted values show reasonable agreement with the simulated values given the uncertainties and sources of noise inherent in our overall approach – the agreement in ${S}_{Y}^{{\rm{displ}}{\rm{.}}}(\theta )$ is nearly exact, while the fitted values of ${S}_{X}^{{\rm{displ}}{\rm{.}}}(\theta )$, for which me made an important simplification, are of the right order of magnitude (we note that greater deviations from simulated values are accompanied by larger error bars). This finding lends support to a hypothesis, offered in Methods below, that redistribution may have a similar form in the wavenumber *q* as does erosion. In particular, it is known that ${S}_{Y}^{{\rm{displ}}{\rm{.}}}(\theta )$ is composed of erosive contributions that are purely negative, and larger contributions from redistribution that are purely positive^31^. Both contributions are required to produce the curves obtained from simulation in Fig. 5b, and the essentially exact agreement between the simulated ${S}_{Y}^{{\rm{displ}}{\rm{.}}}(\theta )$ and the fitted values in Fig. 5a (which are less uncertain than those for ${S}_{X}^{{\rm{displ}}{\rm{.}}}(\theta )$) suggests the presence of both erosion and redistribution in the latter.

## Discussion

For each of the model parameters described above, we found that fits of the extracted dispersion relations to the composite model (4) produced estimates that were in reasonable to excellent agreement with available external simulations, experiments, or theoretical predictions. This finding has several implications. First, it suggests that (4) is a reasonably complete description of the dynamics of the irradiated surface. Indeed, when any one mechanism is omitted from the model, the quality of both the overall fits, and the per-parameter agreement between fits and external estimates, decreases significantly (see Table 1). This is precisely what one would expect given a model that is “as simple as possible, but not simpler.”

**Table 1:** Summary of fit results for different model combinations. The parameters {a, b, d} are defined below in the Methods section.

Model	${{\boldsymbol{\chi }}}_{{\bf{red}}{\boldsymbol{.}}}^{{\bf{2}}}$	*a* [nm]	*b* [nm]	*d* [nm]
Orchard only	225	5.2	9.4	—
Orchard + Bradley	99.6	5.9	0.0	11
Orchard + Norris	71.6	0.0	9.3	—
Orchard + Norris + Bradley	33.2	3.3	3.5	4.4

The first three entries are strictly for comparative purposes; only the final model is discussed in the Results section.

Second, the favorable agreement between the fitted parameter values and available external estimates (e.g. the film thickness) builds confidence in the validity of those fitted values for which external estimates are *unavailable*. Indeed, the fitting process we have employed here can be viewed as the experimental inference of previously-unavailable parameter values. In particular, we have obtained values of the fluidity ${\eta }^{-1}$ and stress parameter *A*, for which no direct experimental measurement strategies currently exist. Ultimately, this approach allows us to overcome a long-standing inability, introduced above, to distinguish physical mechanisms with the same limiting form in the long-wave limit. Here, with access to amplification rates for a much wider range of wavenumbers, such distinctions have become possible.

A straightforward demonstration of this last point can be made in the case of the surface energy relaxation mechanism. Early theoretical models of surface structure formation invoked atomic surface diffusion as the ref. 12, which produces linearized contributions to *R*(*q*) of the form

10 $${R}_{{\rm{Mullins}}}({\bf{q}})=-\,\mu \gamma {|{\bf{q}}|}^{4},$$

where *γ* is again the surface energy, and *μ* is a temperature-dependent mobility. The quartic behavior of this mechanism directly informed the quartic term in the longwave approximation (3); indeed, because the competing Orchard mechanism (11) is also quartic in the longwave limit *q* → 0, it has often been viewed as a “drop-in” replacement for surface diffusion at low operating temperatures. Within the present context, however, we are focused on the opposing limit $q\to \infty$. As described above, in this limit the Orchard mechanism grows linearly in **q**, which is drastically different than the quartic response exhibited by surface diffusion in the same limit. Therefore, the observation in Fig. 1 of nearly linear growth in *R*(*q*) speaks strongly and directly in favor of the Orchard mechanism rather than that of surface diffusion (and is the reason we did not include surface diffusion in the composite model (4)).

A more recent, and still unresolved question on mechanisms concerns the magnitude of the stress effect relative to the prompt collisional effects of erosion and redistribution^21^. In ref. 31, the combination of simulated values of displacement coefficients and an estimate of the strength of surface-confined viscous flow was found to produce good agreement with experimental data on pattern wavelengths for 250 eV Ar^+^ → Si. In ref. 21, the combination of stress and viscous flow was also found to produce good agreement with the data, this time without the need to estimate any parameters. Unfortunately, because these two mechanisms look identical in the limit *q* → 0, it was not previously possible to estimate the relative magnitude of these two effects. Here, by comparing Figs 4a and 5a, we see that the magnitudes of the stress coefficient significantly exceed those of the displacement coefficient at angles below *θ* = 45°. At higher angles, however, erosion seems to play an important role in suppressing the instability in the *x*-direction associated with stress, and driving its own instability in the *y*-direction. This result suggests that a solid understanding of both stress and displacement effects will be required for predictive models of ion irradiation, especially near grazing incidence.

We conclude by summarizing four important findings of this work:

1. With the use of hierarchical fitting methods exploiting the assumed uncorrelated nature of system noise, it is possible to extract much more information from GISAXS analysis of surface roughening than previously reported. In particular, compared to prior studies limited to the range $q\in [0.1,0.4]\,{{\rm{nm}}}^{-1}$, we have obtained values of the linear dispersion relation over the full range of wavenumbers $q\in [0.1,1.5]\,{{\rm{nm}}}^{-1}$ accessible to the x-ray detector in a typical beam line.
2. Although longwave approximations of the dispersion relation associated with ion bombardment are convenient for intuitive theoretical discussions, they are inappropriate for comparison with experimental results containing data at higher values of *q*. Instead, dispersion relations retaining full wavenumber dependence are required to obtain good agreement between experiment and theory. As a demonstration of this point, we found that the high-*q* data are entirely inconsistent with the use of surface diffusion as a surface energy relaxation mechanism, but highly consistent with the use of thin-film viscous flow.
3. The comparison of experimental data across the full range of available wavenumbers, to linear models retaining full wavenumber dependence, enables the extraction of significantly more model parameters than has previously been possible. In particular, we are able to extract reasonable values of (a) the angle-dependent thickness of the amorphous thin film, (b) the ion-enhanced fluidity within that film, (c) the magnitude of the stress being generated by the ion beam, and (d) the strength of prompt atomic displacement mechanisms. In each case the fitted values of these coefficients agreed well with external theoretical, experimental, and simulation results.
4. Most importantly, the approach we have employed enables the evaluation of absolute and relative strength of hypothesized physical mechanisms during ion bombardment. In a direct comparison of the relative magnitudes of the stress mechanism to the combined effects of erosion and redistribution, we find that the former is significantly larger at angles below about *θ* = 60°. Although questions remain regarding the accuracy of BCA for estimating ion-induced displacement effects^33^, the agreement of fits with the BCA in this case strongly supports the possibility that stress may be more important than impact-induced redistribution in the formation of patterns during low-energy ion bombardment of Si, and highlights the value of efforts such as^34, 35^ to understand it in more detail.

However, we believe the most important result we have obtained lies in the methodology itself. The overall quality of the composite model fits both to the underlying experimental data, and to external estimates of individual model parameters, both supports the validity of the overall approach, and also suggests that the models employed here encompass most of the relevant physical mechanisms in operation during ion bombardment. The ability to extract most model parameters of interest directly from experimental data represents a significant advance toward a comprehensive understanding of the ion-induced pattern formation process, and an increase in the value of GISAXS studies more generally.

## Methods

### Experimental Methods

Two sets of data were used in these studies. For both, samples were p-doped Si(001) with resistivity 1–10 ${\rm{\Omega }}\cdot {\rm{cm}}$, affixed clipless to a molybdenum sample platen with extra care to minimize secondary collisions that can lead to sputtering of impurities onto the surface. The sample platen was mounted in a custom vacuum chamber with base pressure 1 × 10^−8^ Torr. Samples were bombarded at room temperature with 1 keV Ar^+^ using a Physical Electronics Inc. PHI ion source with a beam diameter of approximately 1.5 cm.

#### Existing Data

For x-ray experiments with projected incident x-ray beam direction parallel to the projected ion beam direction on the sample (i.e., revealing corrugations perpendicular to the ion beam, which is typically called the y-direction), we re-used measurements taken in refs 4 and 5. These experiments were performed using a dedicated facility for the study of surface and thin film processes on beamline X21 at the National Synchrotron Light Source (NSLS), using a photon energy of 10 keV (0.124 nm wavelength) that was selected by a Si(111) monochromator and a flux of approximately 10^12^ photons/s. The x-ray incidence angle on the sample surface was 0.82 from the surface tangent, and a 384 pixel linear detector measured the scattering pattern with an exit angle set to the critical angle for silicon, 0.18. Samples were approximately 1 × 1 cm^2^ in area and were affixed to the sample platen using silver paste, and irradiated with an ion flux of approximately $2\times {10}^{12}\frac{{\rm{ions}}}{{{\rm{cm}}}^{2}\,\sec }$ reckoned in a plane perpendicular to the ion beam. We collected data at irradiation angles $\theta =\{0,25,45,55,70,80\}$. More details of the experiment can be found in refs 4 and 5.

#### New Data

For x-ray measurements with projected incident x-ray beam direction perpendicular to the ion beam direction (i.e., revealing corrugations parallel to the ion beam, which is typically called the *x*-direction), new experiments were performed at the Cornell High-Energy Synchrotron Source (CHESS) on beamline G3 in a facility that focuses on surface and thin film processes, using a photon energy of 11.2 keV (0.111 nm wavelength) selected by a Si(111) monochromator with a flux of approximately $3\times {10}^{13}\frac{{\rm{photons}}}{{{\rm{mm}}}^{2}\cdot \,\sec }$ and beam dimensions 2 mm × 1 mm. The x-ray incidence angle on the sample surface was 0.89°. Scattered photons were measured by a 497 × 195 pixel PILATUS area detector. Samples were approximately 1.25 × 1.25 in^2^ in order to cover the entire elevated area of the sample platen to further minimize incorporation of metallic impurities, and were affixed using molten indium. The ion flux was approximately $1.2\times {10}^{13}\frac{{\rm{ions}}}{{{\rm{cm}}}^{2}\cdot \,\sec }$ reckoned in a plane perpendicular to the ion beam. We collected data at ion incidence angles $\theta =\{0,20,30,50,65\}$.

#### Discussion

Experimental work at the CHESS synchrotron was informed by the earlier experiments at the NSLS. Analysis of NSLS data suggested that the nonlinear regime of nanopattern amplification may start at lower fluences than previously expected, so experiments at CHESS focused on lower fluence nanopatterning. Before data collection, samples were bombarded under vacuum with a fluence of at least $8.6\times {10}^{15}\frac{{\rm{ions}}}{{{\rm{cm}}}^{2}}$ to remove native oxide, amorphize the surface layer, and minimize initial transient behavior. Samples that had been sitting in atmosphere had a critical GISAXS angle slightly different than that of samples having undergone significant bombardment at vacuum, which we attribute to the removal of the native oxide layer. We ensured that each sample was bombarded in vacuum for at least long enough to cause this shift in critical angle before using the sample for data collection. To ensure amorphization, the chosen initial fluence was further selected to be at least one order of magnitude greater than the amorphization fluences reported for 1 keV Ar^+^ irradiation of Si (111) and Ge (100), which are in the range of $0.9-3.0\times {10}^{14}\frac{{\rm{ions}}}{{{\rm{cm}}}^{2}}$ ^36^. If data were to be collected for an irradiation angle associated with [smoothing/roughening], then oxide was removed at an angle associated with [roughening/smoothing].

However, at CHESS an irregular beam intensity profile, as well as variations in beam position by as much as 200 m over the course of data collection for a single sample, resulted in a variable incident x-ray intensity that did not correspond to any flux values measured upstream. This required scaling scattering patterns by observed central peak intensity rather than measured incident beam intensity, as described in Section A of the Supplemental Material. In addition, the larger sample size and wider beam profile at the CHESS facility resulted in a significant fraction of the scattering signal originating outside the region of maximum ion beam intensity. Hence, although the ion flux at CHESS was nominally higher than the ion flux at NSLS, our recorded intensity profiles strongly suggest that the actual effective flux was lower at CHESS by a factor of about 3.3. To compensate for this, the time scale of all CHESS experiments was decreased by a common factor of 3.3.

### Analytical Methods

The analytical methods used in this manuscript have been made available as an open-source library, called PyGLIDRE – Python GISAXS LInear Dispersion Relation Extraction. This library is described in some detail in Section B of the Supplemental Material; here we summarize the main background and capabilities.

#### Prior Work

As described above, at early times in the pattern formation process, one may assume that the amplitude of surface structures is small, in which case the measured GISAXS intensity corresponds to the surface structure factor *S*(**q**). By performing Fourier analysis on the linear governing Eq. (1), one obtains a solution to the evolution of the structure factor of the form Eq. (2), which is then fit to the measured timeseries *S*(*q*, *t*) for each wavenumber *q* to reconstruct the dispersion relation *R*(*q*). If the x-ray beam is aligned in the same direction as the ion beam, one obtains angle-dependent values of *R*(0, *q*_*y*_), whereas angle-dependent values of *R*(*q*_*x*_, 0) are obtained if the x-ray beam is aligned perpendicular to the ion beam.

**Figure 6:**
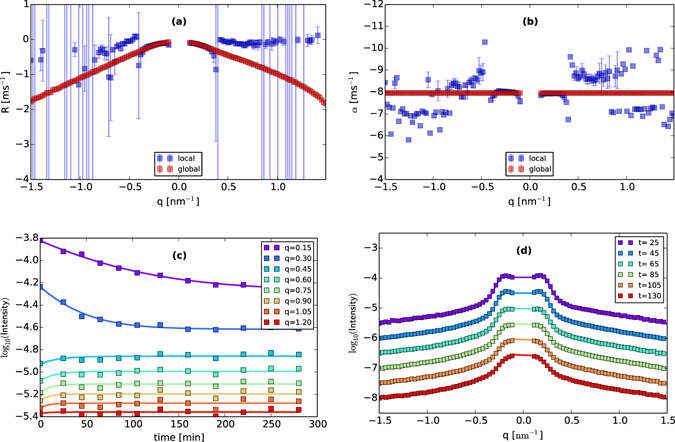
An illustration and evaluation of the hierarchical fitting approach, for irradiation of Si by 1000 eV Ar+ ions, at an angle of incidence of 20 degrees. (**a**) Fitted values of *R*(*q*) for local vs. hierarchical fits. (**b**) Fitted values of *α* for local vs. hierarchical fits. (**c**) Comparison of data slices *I*(*q*_0_, *t*), for selected values of *q*_0_, to hierarchically-fitted values of the same. (**d**) Comparison of data slices *I*(*q*, *t*_0_) for selected values of *t*_0_, to hierarchically-fitted values of the same [to facilitate evaluation, we have plotted every fifth value of *q*, and displaced successive plots downwards by 0.5 units]. These results suggest that the hierarchical fit can return much more information than a local fit, while remaining consistent with the underlying data. We also note that the hierarchical fits of {*R*, *α*} are consistent with the local fits over the range $q\in [0.1,0.3]$ where the local fits produce meaningful results.

However, the analyses performed in refs 4, 5 were limited to a typical range of about $q\in [0.1,0.4]\,{{\rm{nm}}}^{-1}$ for most incidence angles, significantly narrower than the range $q\in [0,1.5]\,{{\rm{nm}}}^{-1}$ available on the detector. The reason is that above a certain value of *q*, the intensity is quite small initially, and almost immediately reaches the (also small) steady value ${I}_{\infty }=-\,\frac{\alpha }{2R}$. For an essentially constant data set, the problem of independently fitting each of the three parameters $\{{I}_{0},R,\alpha \}$ is under-determined, and results in very large variations in *R* and *α* as the minimizer seeks very small reductions in the objective function (see blue squares in Fig. 6a,b, and compare the erratic behavior in (5)b for *q* > 0.4 with the essentially flat timeseries in (6)c for *q* > 0.4). Consequently, prior studies have included values of *R* only for those values of *q* where both *R* and *α* could be independently obtained.

#### Hierarchical Fitting

To overcome this limitation, we here exploit the expected and observed properties of the noise term. If the noise *β*(*q*, *t*) produced by the ion gun is uncorrelated in space and time, as long assumed^37^, then the term *α* should be simply a constant, and indeed, over the range of *q* where the intensity varies enough to independently fit $\{{I}_{0},R,\alpha \}$, the fitted value of *α* does appear to be a constant. Based on this observation, we have explicitly assumed that *α* is a constant, and fitted a single, *global value* of *α* across all values of *q*, while allowing *I*_0_ and *R* to be fitted *locally* for each value of *q*. This is accomplished with a hierarchical fitting strategy described in the Supplemental Material. An example of the result is shown by the red squares in Fig. 6a,b, where we see the improvement in the range of extracted values of *R*(*q*) and *α* attained by the hierarchical fits, noting that those fits are consistent with the strictly local fits when the latter provide meaningful results. Then, in Fig. 6c,d we compare cross-sections of the intensity function *I*(*q*, *t*) to hierarchical fits of the same, finding good agreement both for selected constant values of *q*, and also for selected constant values of *t*, despite the selection of just a single value of *α* for all values of *q*.

### Modeling

We now consider the comparison of the recovered values of *R*(*q*) to various theoretical models. Because our analytical approach yields values over a much wider range of *q* than was previously available, we can see immediately from Fig. 1 that the longwave approximation in Eq. (3) is unlikely to be a good description of *R*(*Q*) over this expanded range. Indeed, the most striking feature of our evaluations of *R*(*q*) is that they grow at most *linearly* as $Q\to \infty$. It is therefore clear that Eq. (3) must be replaced with non-truncated models to attain consistency with the observed behavior of *R*(*Q*) as $Q\to \infty$. (Indeed, attempts to fit Eq. (3) to the data return *negative* values of *B*, a non-physical result that contradicts the explicit assumptions of models generating such terms).

#### Thin-Film viscous flow

We begin by considering the mechanism of surface energy relaxation, which is usually assumed to dominate surface dynamics in the limit $q\to \infty$. The linear behavior of *R*(*q*) in this limit observed in Fig. 1 strongly suggests the mechanism of ion-enhanced viscous flow^19^, in which the ion beam enhances the fluidity of the amorphous layer, which then enables viscous flow in the Stokes limit of high viscosity. Such flow was shown by Orchard to exhibit the dispersion relation^18^

11 $${R}_{{\rm{O}}{\rm{r}}{\rm{c}}{\rm{h}}{\rm{a}}{\rm{r}}{\rm{d}}}({\bf{q}})=-\,\frac{\gamma }{2\eta {h}_{0}}(\frac{Q[\sinh (2Q)-2Q]}{1+2{Q}^{2}+\cosh (2Q)})$$

where ${\eta }^{-1}$ is the fluidity, *h*_0_ is the average amorphous film thickness, and $Q={h}_{0}|{\bf{q}}|$. Although widely known for exhibiting quartic behavior at small wavenumbers ($R\propto {q}^{4}$ as *q* → 0), this mechanism behaves linearly at high wavenumbers ($R\propto q$ as $q\to \infty$). We note that although the Orchard mechanism has recently been favored over e.g. surface-diffusion due to its consistency with studies on temperature dependence of the irradiation process, and the results of molecular dynamics studies^20^, Fig. 1 represents, to our knowledge, the first *experimental* observation of definitively Orchard-like behavior under irradiation conditions.

#### Beam-Induced Stress

Because the Orchard model is unconditionally stable, whereas our data are consistent with a well-known instability at high angles of incidence, we now need to add a mechanism capable of generating an instability, for which a full-spectrum model is available. We first consider two recent models describing the effect of ion beam-induced stress (which can be in the GPa range^27^) on the viscous flow in the film. The first is due to Castro and Cuerno^23^, and has the form

12 $${R}_{{\rm{C}}{\rm{a}}{\rm{s}}{\rm{t}}{\rm{r}}{\rm{o}}/{\rm{C}}{\rm{u}}{\rm{e}}{\rm{r}}{\rm{n}}{\rm{o}}}(q)={f}_{E}{C}_{X,Y}^{{\rm{s}}{\rm{t}}{\rm{r}}{\rm{e}}{\rm{s}}{\rm{s}}}(\theta )\frac{[\sinh (2Q)-2Q]}{Q[1+2{Q}^{2}+\cosh (2Q)]}.$$

Here, *f*_*E*_ describes the characteristic strength of an angle-dependent “effective body force” (EBF), and ${C}_{X,Y}^{{\rm{stress}}}(\theta )$ is a function of angle. The second is due to Norris^21^, and has a dispersion relation with the somewhat different form

13 $${R}_{{\rm{N}}{\rm{o}}{\rm{r}}{\rm{r}}{\rm{i}}{\rm{s}}}(q)=6fA{C}_{X,Y}^{{\rm{s}}{\rm{t}}{\rm{r}}{\rm{e}}{\rm{s}}{\rm{s}}}(\theta )\frac{{Q}^{2}}{[1+2{Q}^{2}+\cosh (2Q)]}$$

where *f* is the ion flux and *A* is the magnitude of an imparted stress-free strain, and again ${C}_{X,Y}^{{\rm{stress}}}(\theta )$ is a function of angle. As discussed in ref. 21, the models differ in the way stress is introduced, leading to the different forms for the first terms in Eqs (12) and (13) (the second term in each is again the Orchard relaxation rate). In principle, the models could be distinguished experimentally through the polynomial versus exponential decay rates as $Q\to \infty$, which arise due to different modeling assumptions. Unfortunately this distinction is more subtle than that existing between surface diffusion and surface-confined viscous flow, and because the models have the same behavior for small values of *Q*, both yield about the same fit of the existing data. For the reasons discussed in ref. 21, we will use Eq. (13) to perform the fits.

#### Prompt Atomic Displacements

The fitted value of *R*(*q*_*x*_, 0) for *θ* = 80° is unique among the data sets in that it appears to asymptote to a non-zero constant as $q\to \infty$. This behavior is not compatible with either of the previous mechanisms. Suggestively, this angular and directional regime is precisely that in which erosive effects have been presumed to be most important^21, 31^. Therefore, we now consider the addition of the Sigmund model of atomic sputtering^7, 8^, for which the exact dispersion relation was recently obtained by Bradley^22^:

$$\begin{array}{rcl}{R}_{{\rm{Bradley}}}({q}_{x},{q}_{y}) & = & \frac{{\rm{\Lambda }}J\varepsilon {a}^{4}}{\sqrt{2\pi }{(\alpha \beta \sqrt{{B}_{1}})}^{3}}\exp [-\frac{{a}^{4}{\cos }^{2}\theta }{2{\alpha }^{2}{\beta }^{2}{B}_{1}}]\\  &  & \times [{\cos }^{2}(\theta )-\exp (-\frac{{a}^{2}}{2{B}_{1}}{q}_{x}^{2}-\frac{{\beta }^{2}}{2}{q}_{y}^{2}+\frac{{a}^{3}\,\sin (\theta )}{{\alpha }^{2}{B}_{1}}i{q}_{x})\\  &  & \times ({\cos }^{2}(\theta )-\frac{{\beta }^{2}\,\sin (\theta )}{a}i{q}_{x})]\end{array}$$

where ${\rm{\Lambda }}J\varepsilon$ is a proportionality constant, *a* is the mean ion penetration distance, *α* and *β* are the longitudinal and lateral straggles, and ${B}_{1}={(\frac{a}{\alpha })}^{2}\,{\sin }^{2}(\theta )+{(\frac{a}{\beta })}^{2}\,{\cos }^{2}(\theta )$ is a geometrical constant.

When using this result to help fit our extracted values of *R*(*q*), we will make two important assumptions. First, for simplicity, we shall abbreviate this result to the following form

14 $${R}_{{\rm{Bradley}}}(q)\approx {C}_{X,Y}^{{\rm{displ}}{\rm{.}}}(\theta )(1-\exp (-\frac{1}{2}{(Dq)}^{2}))$$

where constants and most of the angle dependence are absorbed into the function ${C}_{X,Y}^{{\rm{displ}}{\rm{.}}}(\theta )$, and *D* is a characteristic lengthscale of the collision cascade. This abbreviation is exact in the *q*_*y*_-direction (*q*_*x*_ = 0) if *α* = *β*. In the *q*_*x*_-direction, or if $\alpha \ne \beta$, the abbreviation is inexact, but nevertheless exhibits the same general dependence on the wavenumber (a Gaussian transition from 0 to 1) while allowing all angle dependence to be represented by the fit parameter ${C}_{X,Y}^{{\rm{displ}}{\rm{.}}}(\theta )$.

Second, although ref. 22 considered only the Sigmund model of erosion, we here hypothesize that the accompanying collisional mechanism of mass redistribution^1, 10^, though exhibiting a markedly different dependence on irradiation angle^4, 11, 31^, nevertheless exhibits a similar dependence on the wavenumber *q*. This is done in part by necessity – existing models for redistribution are inherently longwave in nature, as no true analog of Sigmund’s model for erosion exists for redistribution (see discussion in ref. 30). However, erosion and redistribution are both, ultimately, caused by the same collision cascade, are occasionally both modeled using rotating Gaussian ellipsoids (see refs 11 and 38), and the expansion of the full collision cascade in moment form reveals that each erosive term has a redistributive counterpart^30, 31^. Therefore the assumption of similar dependence on q has a reasonable physical and theoretical basis, in addition to the statistical support observed in Figure 5.

#### Fit Constraints

The combined model exhibits five free parameter groups per angle: the film thickness *h*_0_, the effective fluidity $\gamma {\eta }^{-1}$, a measure of beam stress $fA{S}_{X,Y}^{{\rm{stress}}}(\theta )$, a measure of prompt displacement effects ${S}_{X,Y}^{{\rm{displ}}{\rm{.}}}(\theta )$, and a lengthscale *D* associated with the prompt effect. However, we found that attempting to fit each of these parameters independently for each angular data set was problematic. In particular, the appearance of *h*_0_ in the denominator of the leading coefficient of the Orchard term couples the values of *h*_0_ and $\gamma {\eta }^{-1}$, allowing the minimizer to pursue small decreases in the residual – without changing the relative magnitude of the effect – by moving these parameters in opposing directions. A similar coupling behavior is seen between the coefficients ${S}_{X,Y}^{{\rm{displ}}{\rm{.}}}(\theta )$ and *D*.

To limit large fluctuations in the fitted values of these parameters, we again used a hierarchical fitting scheme, allowing $\gamma {\eta }^{-1}$, *fA*, and *C* to be fitted independently for each angle, but requiring *h*_0_ and *D* to be a global functions of the form

15 $${h}_{0}(\theta )=a+b\,\cos (\theta ).$$

16 $$D(\theta )=d$$

These forms represent the leading terms of a Fourier cosine series, chosen because of assumed symmetry of these parameters in the irradiation angle. Applying this overall fitting strategy, we obtain the fitted versions of Eq. (13) displayed in Fig. 1. We see that the fitted models match quite closely the extracted values of *R*(*q*). For completeness, we have also fitted the data to various subsets of the mechanisms just described. These results are summarized in Table 1, showing that all three mechanisms contribute significantly to the minimization of modified ${\chi }^{2}$ error.

## Electronic supplementary material

Supplementary Information

## References

[1] Moseler M, Gumbsch P, Casiraghi C, Ferrari AC, Robertson J (2005). The ultrasmoothness of diamond-like carbon surfaces. Science.

[2] Chan WL, Chason E (2007). Making waves: kinetic processes controlling surface evolution during low energy ion sputtering. J. Appl. Phys..

[3] Cross, M. & Greenside H. *Pattern Formation and Dynamics in Nonequilibrium Systems*. ISBN: 0521770505 (Cambridge University Press, 2009).

[4] Madi CS, Anzenberg E, Ludwig KF, Aziz MJ (2011). Mass redistribution causes the structural richness of ion-irradiated surfaces. Phys. Rev. Lett..

[5] Anzenberg E, Madi CS, Aziz MJ, Ludwig KF (2011). Time-resolved measurements of nanoscale surface pattern formation kinetics in two dimensions on ion-irradiated si. Physical Review B.

[6] Mutzke, A., Schneider, R., Eckstein, W. & Dohmen, R. SDTrimSP version 5.0. IPP Report 12/8, Garching (2011).

[7] Sigmund P (1969). Theory of sputtering. I. Sputtering yield of amorphous and polycrystalline targets. Phys. Rev..

[8] Sigmund P (1973). A mechanism of surface micro-roughening by ion bombardment. J. Mater. Sci..

[9] Bradley RM, Harper JME (1988). Theory of ripple topography induced by ion bombardment. J. Vac. Sci. Technol..

[10] Carter G, Vishnyakov V (1996). Roughening and ripple instabilities on ion-bombarded si. Phys. Rev. B.

[11] Davidovitch BP, Aziz MJ, Brenner MP (2007). On the stabilization of ion sputtered surfaces. Phys. Rev. B.

[12] Mullins WW (1959). Flattening of a nearly plane solid surface due to capillarity. J. Appl. Phys..

[13] Volkert CA (1991). Stress and plastic flow in silicon during amorphization by ion bombardment. J. Appl. Phys..

[14] Snoeks E, Weber T, Cacciato A, Polman A (1995). MeV ion irradiation-induced creation and relaxation of mechanical stress in silica. J. Appl. Phys..

[15] Brongersma ML, Snoeks E, van Dillen T, Polman A (2000). Origin of MeV ion irradiation-induced stress changes in SiO_2_. Journal of Applied Physics.

[16] Mayr SG, Averback RS (2005). Ion-irradiation-induced stresses and swelling in amorphous ge thin lms. Phys. Rev. B.

[17] Chan WaiLun, Chason Eric (2008). Stress evolution and defect di usion in cu during low energy ion irradiation: Experiments and modeling. J. Vac. Sci. Technol. A.

[18] Orchard SE (1962). On surface levelling in viscous liquids and gels. Appl. Sci. Res..

[19] Umbach CC, Headrick RL, Chang K-C (2001). Spontaneous nanoscale corrugation of ion-eroded SiO_2_: The role of ion-irradiation-enhanced viscous flow. Phys. Rev. Lett..

[20] Vauth S, Mayr SG (2007). Relevance of surface viscous flow, surface di usion, and ballistic effects in kev ion smoothing of amorphous surfaces. Phys. Rev. B.

[21] Norris, S. A. Stress-induced patterns in ion-irradiated silicon: model based on anisotropic plastic flow. *Phys*. *Rev*. *B* **86**, 235405, arxiv:1207.5754 (2012).

[22] Bradley RM (2011). Exact linear dispersion relation for the sigmund model of ion sputtering. Physical Review B.

[23] Mario Castro, Rodolfo Cuerno (2012). Hydrodynamic approach to surface pattern formation by ion beams. Applied Surface Science.

[24] Hofsäss H, Zhang K, Pape A, Bobes O, Brötzmann M (2012). The role of phase separation for self-organized surface pattern formation by ion beam erosion and metal atom co-deposition. Appl. Phys. A.

[25] Nietiadi ML, Sandoval L, Urbassek HM, Möller W (2014). Sputtering of Si nanospheres. Physical Review B.

[26] Scott, A. Norris. Pycraters: A python framework for the collection of crater function statistics. arXiv:1410.8489.

[27] Madi, C. S.. *Linear Stability and Instability Patterns in Ion Bombarded Silicon Surfaces*. PhD thesis (Harvard University, 2011).

[28] Eaglesham DJ, White AE, Feldman LC, Moriya N, Jacobson DC (1993). Equilibrium shape of Si. Phys. Rev. Lett..

[29] Ishii, Y., Madi, C., Aziz, M. J. & Chason, E. Stress evolution in si during low-energy ion bombardment. *Journal of Materials Research* (2014).

[30] Norris SA, Brenner MP, Aziz MJ (2009). From crater functions to partial differential equations: A new approach to ion bombardment induced nonequilibrium pattern formation. J. Phys. Cond. Matt..

[31] Norris SA (2011). Molecular dynamics of single-particle impacts predicts phase diagrams for large scale pattern formation. Nature Communications.

[32] Harrison Matt P, Bradley RMark (2014). Crater function approach to ion-induced nanoscale pattern formation: Craters for flat surfaces are insuffcient. Physical Review B.

[33] Bukonte L (2013). Comparison of molecular dynamics and binary collision approximation simulations for atom displacement analysis. Nuclear Instruments and Methods in Physics Research B.

[34] Castro M, Gago R, Vázquez L, Muñoz-García J, Cuerno R (2012). Stress-induced solid flow drives surface nanopatterning of silicon by ion-beam irradiation. Physical Review B.

[35] Moreno-Barrado, A. *et al*. Nonuniversality due to inhomogeneous stress in semiconductor surface nanopatterning by low-energy ion-beam irradiation. *Physical Review B* (2015).

[36] Bock W, Gnaser G, Oechsner H (1993). Modification of crystalline semiconductor surfaces by low-energy Ar^+^ bombardment: Si(111) and Ge(100). Surface Science.

[37] Cuerno R, Barabási A-L (1995). Dynamic scaling of ion-sputtered surfaces. Phys. Rev. Lett..

[38] Liedke, B. *Ion beam processing of surfaces and interfaces*: *Modeling and atomistic simulations*. PhD thesis (Helmholtz Zentrum Dresden Rossendorf, 2011).

