# Supplementary material for: Distinguishing physical mechanisms using GISAXS experiments and linear theory: the importance of high wavenumbers
Source: Sci Rep. 2017 May 17;7:2016. doi: 10.1038/s41598-017-01059-x (PMC5435725; doi:10.1038/s41598-017-01059-x)
Supplement: Supplementary file 1 — Supplementary Information [file 41598_2017_1059_MOESM1_ESM.pdf]

Supplement to "Distinguishing physical mechanisms using GISAXS experiments and linear theory: the importance of high wavenumbers"

Scott A. Norris[1], Joy C. Perkinson[2], Mahsa Mokhtarzadeh[3], Eitan Anzenberg[3], Michael J. Aziz[2], and Karl F. Ludwig, Jr.[3,4]

August 9, 2016

[1]Department of Mathematics, Southern Methodist University, Dallas Texas 75275, USA
[2]Harvard School of Engineering and Applied Sciences, Cambridge Massachusetts 02138, USA
[3]Department of Physics, Boston University, Boston Massachusetts 02215, USA
[4]Division of Materials Science and Engineering, Boston University, Boston Massachusetts 02215, USA

A Additional Experimental Methods

Here we describe in more detail our response to several challenges to data collection and analysis.

For experiments measuring structures formed under ion irradiation, care must be taken to ensure that no impurities are incorporated from sample holders, chamber walls, or other nearby structures with line of sight to the sample. Impurities can be minimized by coating nearby parts of the chamber with material unlikely to affect pattern formation; in these experiments, for example, large pieces of silicon were used to cover metallic components of the chamber.

The surface oxide layer was removed from samples *in situ*. It is advisable to remove the surface oxide layer under different environmental conditions than those to be used for the experiment – for instance, if collecting data for an incidence angle expected to exhibit roughening, the oxide layer can be removed for an incidence angle expected to exhibit smoothing. This increases the likelihood that the initial and final measured intensity profiles will be different enough to extract the rate of decay from the former to the latter.

At the CHESS G3 station, the beam shifts position by a few hundreds of microns over time. Although the intensity of the full incident beam is measured before the sample, the beam is inhomogeneous and only part of it hits the sample at low incidence angles. To normalize the scattered intensity, the height of the central peak was used as a calibration. In general, the intensity of the central peak can change based on surface structures, so this technique is not ideal, but it can be used as a first pass in situations where surface structure changes are small and no better measure of photon flux variations is available.

At high wavenumbers the surface scattering is inherently small, and time-dependent scattering background can obstruct accurate measurements at high wavenumbers. One approach to removing this background would be to sample the intensity of the x-ray background a two-dimensional detector in the nominally dark region of the detector, away from the GISAXS pattern. However, desiring an approach that would also be applicable to our data obtained with a 1D detector, we instead removed variations – from an exponential fit – in the average intensity of the high-wavenumber regions, as described in Appendix B

Although high wavenumbers provide the best opportunity to distinguish competing physical mechanisms, significant obstacles inhibit the collection of clean high-q data. These obstacles arise because the growth rate $R\left(q\right)$ usually reaches its most negative values at the highest values of q. Consequently, the observed high-q intensities are small at all times, and the transitions between initial and final intensities occur rapidly. Thus the fitting at high wavenumbers is enabled primarily by the assumption of constant α, and is sensitive to the final structure factor.

B Data Analysis with PyGLIDRE

In this section we describe in more detail the technical aspects of our analytical methods, which we have released as an open-source library PyGLIDRE: Python GISAXS Linear DIspersion Relation Extraction.

Extraction and Preparation. We assume that after experiment, the user has obtained 2D intensity image files, as well as a configuration file. The first step is to read these files, and store the results in a common format describing the sampled times t and wavenumbers q, and the intensity $I\left(q,t\right)$. This requires a wrapper for specifying experimental setup and reading instrumental output, averaging multiple images taken at the same fluence, and so on. After removing any "hot" or "cold" pixels (pixels that report an erroneously large or small number of photons) using standard image processing techniques, we then must identify the location of the Yoneda peak. If the sensor was suitably positioned and shielded, this may simply be the

pixel in each image with the highest intensity. Otherwise, it will be a local maximum, in which case the user should supply a rectangular subset inside of which the Yoneda peak is the maximum.

Normalization. The next step is to normalize each image to a common scale. A primary source of noise involves an apparent variation in the number of photons striking the sample. In principle these photons are counted by a detector before entering the chamber, and recorded in the facility output. Each wrapper within the extraction section should include a way to load the photon count associated with each image, so that normalization is as simple as dividing the resulting array by the photon count. In cases where the photon count recorded by the facility is deemed not to accurately reflect the number of photons scattered from the sample, one can in theory exploit the expected property of vertical translation invariance present in many free-surface problems. If a system is vertically translation invariant, then only *derivatives* of the height field can appeear in the governing equation – terms of the form $h_t = R_0 h$, are excluded, implying that $R(q) \to 0$ as $q \to 0$. Because the GISAXS intensity exhibits large peaks at $q = 0$, and these peaks should not change over time in the linear regime, scaling by the peak height should in principle be valid in the linear regime if the photon count is unavailable or unreliable. We applied the scaling

$$I(q,t) \to I(q,t) \,/ \int_{-.03}^{+.03} I(q,t)\; dq.$$

This process is illustrated in Figure (1)a,b, where it is observed to eliminate fluctuations in the peaks over time.

Smoothing. In all our experimental work, we found that while a suitable normalization strategy produced peak heights evolving according to theoretical expectations, intensity levels in the tails still exhibited considerable variation in time. These variations were nearly uniform across wavenumbers q, apparently ruling out shot noise (which would be expected to be uncorrelated in wavenumber). Further examination of available data suggested the presence of an additive "background" scattering caused by some feature of the experimental setup. To filter out these variations, we find the average intensity of data points in the tails, fit the resulting timeseries to an exponential function, and then subtract the difference between fit and average from the data:

$$I(q,t) \to I(q,t) - \left\{ \langle I(q,t) \rangle_{q=1.2}^{q=1.5} - \mathcal{F}\left[\langle I(q,t) \rangle_{q=1.2}^{q=1.5} \right] \right\}$$

where $\langle \square \rangle$ denotes averaging in q, and $\mathcal{F}[\square]$ indicates the fitted timeseries of the result. This process is illustrated in Figure (1)a,b, where it is observed to significantly reduce fluctuations in the locations of the tails over time.

Fitting via LMFIT. As a result of the previous processes, we have now obtained a reasonably smooth function $I(q,t)$ describing the intensity of the structure factor as a function of both wavenumber q and time t. As described in the main text, this is then fit to the function

$$\langle S(q,t) \rangle = \left[S(q,0) + \frac{\alpha}{2R(q)} \right] \exp\left(2R(q)\,t\right) - \frac{\alpha}{2R(q)}$$

Fits are performed using the Python library LMFIT [1], which presents a powerful, accessible implementation of the Levenberg-Marquardt minimization algorithm. This library offers several user-friendly features, such as allowing the specification of minimum and maximum allowable fitted values for each parameter, and

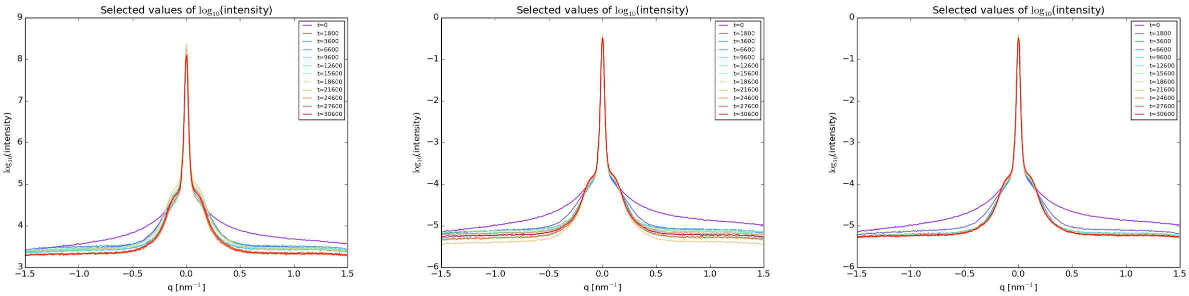

Figure 1: An illustration of noise-removal techniques. (a-c) Plots of $I(q, t_0)$ for selected t_0 at various stages of post-processing: (a) as recorded from the detector, (b) after scaling to account for multiplicative noise, and (c) after removing additive noise observed in the tails. For illustration, we have used data collected at CHESS under irradiation at 80 degrees, which exhibited the most dramatic improvement after these procedures.

automatically returning the uncertainty associated with each parameter in a fit. This last capability is especially important, because the fits of $R(q)$ to the experimental data are used, in turn, as "data" to which theoretical models of the dispersion relation are fit.

Hierarchical fitting with global parameters. As described in the Methods section of the main text, limitations in prior analyses are overcome by assuming the parameter α is constant for all values of q (i.e., we assume white noise in the original PDE governing surface height evolution). The simultaneous identification of a single globally-fitted α and hundreds of locally-fitted pairs $\{I_0, R\}$ is accomplished using a hierarchical fitting strategy, with "outer" and "inner" components. The job of the outer component is simply to find an optimal value of the *global* constant α, which is performed using LMFIT. However, within the objective function used to optimize α, we have inner components to optimize I_0 and R individually for *each value* of q; in these inner optimizations, α is treated as a constant with value equal to its current estimate in the outer loop.

References

[1] Matthew Newville, Till Stensitzki, Daniel B. Allen, and Antonino Ingargiola. Lmfit: Non-linear least-square minimization and curve-fitting for python. *Zenodo*, 2014.
